# Morphology of the Mouthparts of Ladybeetle *Vibidia duodecimguttata* (Coleoptera: Coccinellidae), with Emphasis on Their Sensilla

**DOI:** 10.3390/insects15110854

**Published:** 2024-10-31

**Authors:** Long Chen, Yaping Shi, Ke Wang, Yuanxing Sun, Yanan Hao

**Affiliations:** Biocontrol Engineering Laboratory of Crop Diseases and Pests of Gansu Province, College of Plant Protection, Gansu Agricultural University, Lanzhou 730070, China; chenlong200005@126.com (L.C.); starry1219@163.com (Y.S.); wang_ke00@126.com (K.W.); sunyx1988@126.com (Y.S.)

**Keywords:** mouthparts, sensilla, *Vibidia duodecimguttata*, scanning electron microscopy

## Abstract

Mouthparts are the feeding organs of insects, which play vital roles in the feeding process. The morphology of insect mouthparts has greatly evolved according to different foods and feeding habits. Therefore, studying the structure of the mouthparts is the key to understanding their feeding mechanisms. *Vibidia duodecimguttata* is an important biological control agent for powdery fungus. In this study, the fine morphology of the mouthpart of *V. duodecimguttata* was studied by scanning electron microscopy. The basic components of mouthpart of *V. duodecimguttata* are similar to those of other ladybeetles, while the specific morphology of each part is different. In total, six kinds of sensilla were identified on mouthpart, and twelve types of sensilla were divided according to their morphology, length and distribution. In addition, two kinds of glandular structures were also found, namely perforated plates and cuticular pores. Finally, the morphological differences between mycetophagous and other feeding habits ladybeetles were compared, and the function of each kind of sensilla was discussed, which may establish a solid foundation for further understanding of the feeding mechanism of ladybirds.

## Simple Summary

Mycetophagous ladybeetles feed on powdery mildew fungi throughout their lives and have certain biological control effects. In this study, the fine morphology of the mouthpart of *Vibidia duodecimguttata* and various types of sensilla were investigated in detail using the scanning electron microscopy. Morphological differences with other species were compared and the function of sensilla was discussed.

## 1. Introduction

Coccinellidae is a globally distributed family with a high diversity in ecology, morphology, behavior, and diet [1]. At present, Coccinellidae have been reported to have three feeding habits: phytophagous, predatory, and mycetophagous [2]. Phytophagous ladybeetles that feed on leaves are considered as agricultural pests, while mycetophagous ladybeetles that feed on fungi [3,4], as well as predatory ladybeetles that prey on aphids [5], whiteflies [6], and mites [7], are excellent biological control agents that play an important role in protecting crops and maintaining ecological balance [8]. It has been reported that insect mouthparts have exhibited various forms to adapt to different feeding habits [9]. Hence, studying the morphology of the mouthparts may provide better assistance in understanding their feeding mechanisms.

The morphology of predatory insect mouthparts is always closely related to different foods and feeding habits [10]. For example, the nectar-feeding insects of Meloidae have developed proboscis-like mouthparts specifically adapted for the purpose of nectar consumption [11]. By examining the mouthpart morphology and sensilla types of predatory insects, we can not only understand their feeding mechanism, but also establish a foundation for subsequent biocontrol strategies [12]. So far, a large number of Coleoptera insects have been extensively characterized in terms of their morphology and ultrastructure of mouthparts, including Coccinellidae [13], Bruchinae [14], Curculionoidea [15], Meloidae [11], Scarabaeidae [16], Nitidulidae [17], and Cerambycidae [18]. Most of these insects are phytophagous and predatory species [19,20]. In contrast, limited research has been conducted on mycetophagous beetles, particularly regarding the mouthparts of mycetophagous ladybeetles [21].

*Vibidia duodecimguttata* (Poda, 1761) is a mycetophagous ladybird beetle species that exhibits a wide distribution in the Palearctic region [22,23]. It serves as an obligate consumer of various powdery mildew fungi throughout its life stages [24]. Moreover, experiment has provided evidence for the overwintering advantage of *V. duodecimguttata*, characterized by relatively low winter mortality [21]. In conclusion, this species exhibits significant potential as a biological control agent [25,26]. This study aims to elucidate the fine morphology of the mouthparts of *V. duodecimguttata* and describe the morphological character, number, and distribution of various kinds of sensilla on these mouthparts using scanning electron microscopy (SEM). The results hold significant implications for inferring feeding mechanism.

## 2. Materials and Methods

### 2.1. Insect Collection

Adults of *Vibidia duodecimguttata* were collected from Lanzhou, Gansu Province, China, on 7 July 2022. After collection, specimens were stored in 75% ethanol solution in 4 °C refrigerator before use.

### 2.2. Scanning Electron Microscopy

Ten female and ten male ladybeetles were separately placed in 5 mL centrifuge tubes with 75% ethanol, and then washed twice with an ultrasonic cleaner (SB-5200DTD, Scientz, Ningbo, China) for twenty seconds at a time. After that, there heads were dissected from the bodies under a stereomicroscope (Stemi 305, Zeiss, Suzhou, China), and then were cleaned; and they dehydrated with ethanol at concentrations of 80%, 85%, 90%, and 95%, respectively, for 20 min, and then dehydrated twice in 99.9% ethanol. After dehydration, the mouthparts were placed individually in a clean Petri dish and sufficiently dried in an electrically heated thermostatic drying oven (GZX-GF101-2-BS-II/H, Hengzi, Shanghai, China) at 40 °C for 12 h. Each part of the mouthparts was dissected and mounted on aluminum stubs with double-sided copper sticky tape, and then sprayed with gold using a high-resolution sputter coater (ACE600, Leica, Vienna, Austria). Fine morphology of the mouthpart was observed and photographed with a scanning electron microscope (S3400N, Hitachi, Tokyo, Japan) at 5 kv.

### 2.3. Image Processing and Data Analysis

Images were combined and data were measured with Adobe Photoshop CC 2019 (Adobe Systems, San Jose, CA, USA). The length and diameter of this sensillum were determined from a minimum of ten sensilla of the same type from different position and different specimens. Various types of sensilla were classified and identified according to the external morphology, length, and distribution [27,28].

## 3. Results

### 3.1. Gross Morphology of the Mouthparts

**Figure 1:**
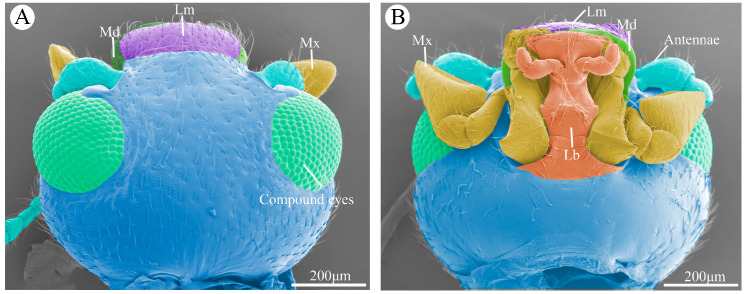
The overall morphology of the head of *Vibidia duodecimguttata*. (**A**) Dorsal view of the head showing the position and morphology of the compound eyes, labrum (Lm), mandible (Md), and maxillae (Mx). (**B**) Ventral view of the mouthparts showing the position and morphology of antennae, labrum (Lm), mandible (Md), maxillae (Mx), labium (Lb).

The mouthparts of *Vibidia duodecimguttata* adults were typical chewing mouthparts, which were composed of a labrum, two symmetrical mandibles, two symmetrical maxillaes, a labium, and a soft hypopharynx. Hypopharynx was a none-sclerotized structure and not visible externally. This study focuses only on the morphology of the sclerotized structures. There was no significant difference in mouthpart morphology between females and males except for that the female mouthparts were slightly larger than that of males, so the subsequent figures and the data were mainly based on the females. In dorsal view, only part of the mouthpart can be seen in front of the head, including the whole labrum, part of the maxillary palp and part of the mandibles (Figure 1A). In ventral side, almost all structures of mouthpart can be seen (Figure 1B).

### 3.2. Types and Morphology of Sensilla on Mouthparts

In total, six kinds of sensilla were distinguished on the mouthparts of *V. duodecimguttata*, including two types of sensilla chaetica (Sch), four types of sensilla basiconica (Sb), two types of sensilla styloconica (Sty), one types of sensilla campaniformia (Sca), two types of sensilla coeloconica (Sco), and one type of sensilla placodea (Sp). Moreover, two kinds of glandular structures were identified, namely cuticular pores (Cp) and perforated plates (Pp) (Table 1).

**Figure 2:**
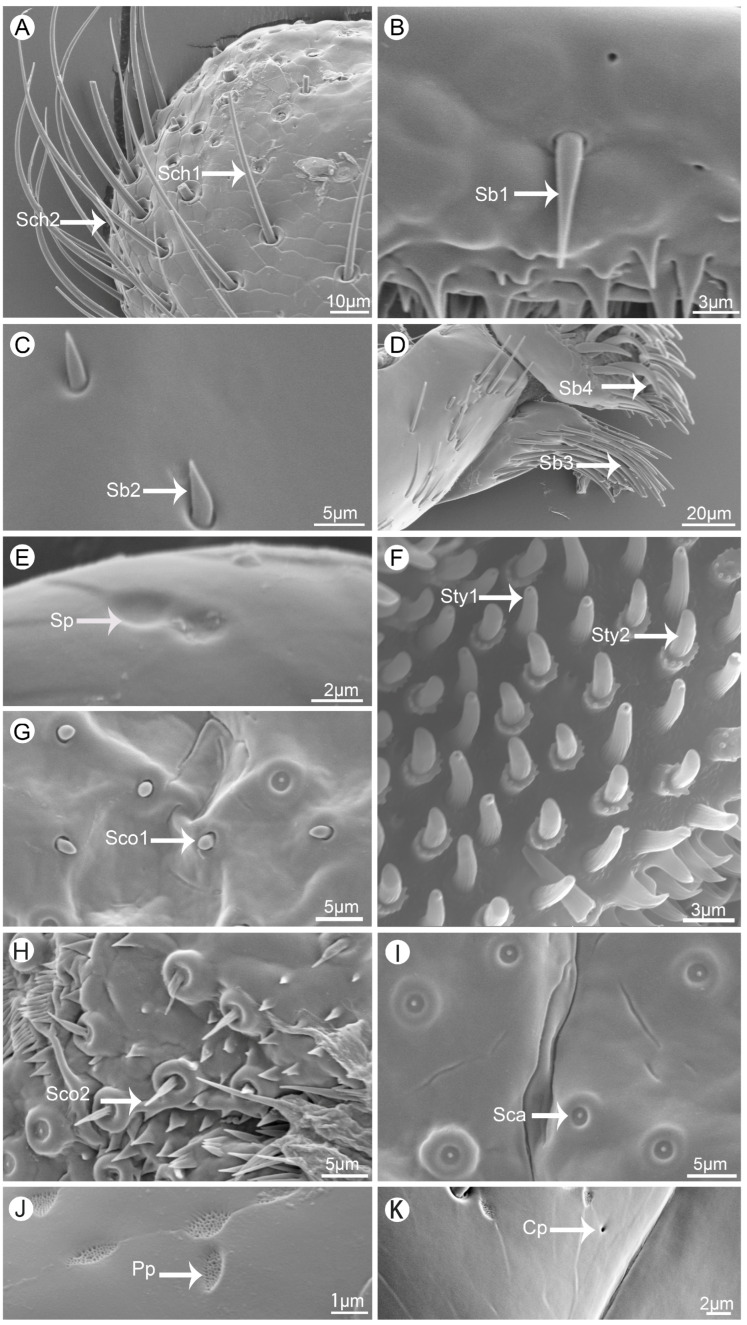
Scanning electron micrographs of different kinds of sensilla and glandular structures on the mouthparts of *Vibidia duodecimguttata*. (**A**) Sensilla chaetica I (Sch1) and sensilla chaetica II (Sch2); (**B**) Sensilla basiconica I (Sb1); (**C**) Sensilla basiconica II (Sb2); (**D**) Sensilla basiconica III (Sb3) and sensilla basiconica IV (Sb4); (**E**) Sensilla placodea (Sp); (**F**) Sensilla styloconica I (Sty1) and sensilla styloconica II (Sty2); (**G**) Sensilla coeloconica I (Sco1); (**H**) Sensilla coeloconica II (Sco2); (**I**) Sensilla campaniformia (Sca); (**J**) Perforated plates (Pp); (**K**) Cuticular pores (Cp).

Sensilla chaetica (Sch) are spiniform with longitudinally groove and no pore on the surface. They are inserted into a round socket. According to the length and shape, they are divided into two types. Sensilla chaetica I (Sch1) are upright with sharp tips (Figure 2A). This type of sensilla is widely distributed on all mouthpart surfaces (Table 1). The length of these sensilla is up to 59.6 μm. Sensilla chaetica II (Sch2) are much longer than type I and the longest one is about 115.2 μm. Their tips are slightly curved (Figure 2A). They can be found on the surface of labrum, labium, and maxillae (Table 1).

Sensilla basiconica (Sb) are conical with a stout base inserted into a round socket, and their surfaces are quite smooth. This kind of sensillum can be divided into four types. Sensilla basiconica I (Sb1) and Sensilla basiconica II (Sb2) are straight with sharp tips (Figure 2B,C), and Sb1 are much longer than Sb2. Sb1 are widely distributed on labrum, maxillae, and labium (Table 1). Sb2 are often distributed on mandibles and labial palpi (Table 1). Sb2 are the shortest among these four types. Sb3 are the longest type, and their length is up to 50.8 μm. Their tips are thin and slightly curved (Figure 2D). Sb3 often gather together on fixed position on lacinia of maxillae. The morphology of Sb4 is quite different from other three types, since they are falcate with convex socket (Figure 2D). Their location is quite fixed on galea of maxillae (Table 1).

Sensilla styloconica (Sty) can be divided into two types, and the morphology of these two types are quite different. Sensilla styloconica I (Sty1) are conical with convex sockets, and their surface are covered with longitudinal grooves. The terminal part is covered with micro-digitations and have an obvious terminal pore (Figure 2F). This type of sensilla is densely distributed on the maxillary palpi and labial palpi (Table 1). Sensilla styloconica II (Sty2) are cylindrical, with longitudinal grooves on the surface and an obvious convex cylindrical socket at the base, which is surrounded by a ring of globular processes (Figure 2F). They are slightly longer (2.8–3.7 μm) than Sty1 while their socket diameters (0.6 to 1.3 μm) are similar to Sty1. They are only distributed on maxillary palpi (Table 1).

Sensilla campaniformia (Sca) are bell-shaped, convex on the outside, concave in the center, and their surfaces are smooth with a small round protuberance in the middle (Figure 2I). They have fixed position and are distributed on epipharynx (Table 1).

Sensilla coeloconica (Sco) can be divided into two types. Sensilla coeloconica I (Sco1) are shorter than Sensilla coeloconica II (Sco2). These two types of sensilla are only distributed on epipharynx (Table 1). Sco2 are sharp while Sco1 are blunt. The round sockets of Sco1 are more obvious than that of Sco2 (Figure 2C,H).

Sensilla placodea (Sp) are circular and slightly concave, and their surfaces are smooth with no pore (Figure 2G). They are quite small, and their diameters are about 2 μm. They have fixed position and are distributed on the first segment of labial palpi (Table 1).

### 3.3. Glandular Structures on Mouthparts

Perforated plates (Pp) are concave structure with many small holes on the smooth surface (Figure 2J), with a variety of irregular shapes, such as round, oval, diamond shaped and so on. They are widely distributed on the surface of all mouthpart structures (Table 1).

Cuticular pores (Cp) are generally round and concave hole on the surface (Figure 2K). They are quite small, and their diameter are less than 1 μm. They can be found on all structures of the mouthpart (Table 1).

### 3.4. Labrum

**Figure 3:**
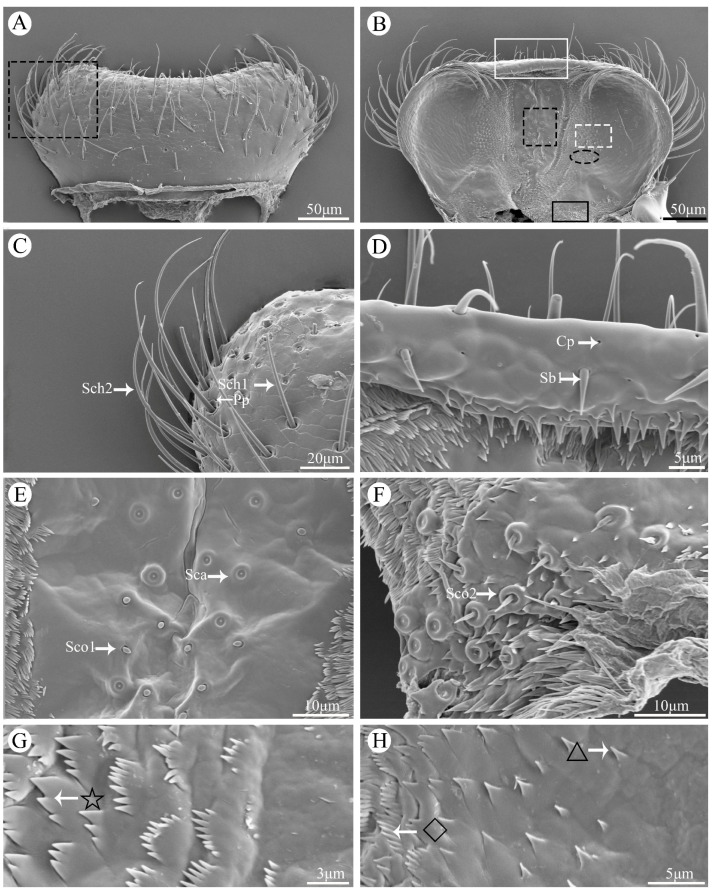
Scanning electron micrographs of the labrum of *Vibidia duodecimguttata*. (**A**) Dorsal view of labrum; (**B**) Ventral view of labrum; (**C**) Enlarged view of black dashed box in (**A**); (**D**) Enlarged view of white solid box in (**B**); (**E**) Enlarged view of black dashed box in (**B**); (**F**) Enlarged view of black solid box in (**B**); (**G**) Enlarged view of white dashed box in (**B**); (**H**) Enlarged view of black ellipse dashed box in (**B**). Sch1: sensilla chaetica I; Sch2: sensilla chaetica II; Pp: perforated plates; Sb1: sensilla basiconica I; Cp: culticular pores; Sca: sensilla campaniformia; Sco1: sensilla coeloconica I; Sco2: sensilla coeloconica II; black pentagram: palmate processes; black rhombic: spiny processes; black triangles: coniform processes.

The labrum is a bilaminar structure that is attached to the anterior edge of the anteclypeus (Figure 3A,B). It has a wide and short shape, almost oblong in appearance. The outer surface exhibits an irregular texture with abundant sensilla and glandular structures, including Sch1, Sch2, Sb1, Pp, and Cp (Figure 3C,D). On the other hand, the inner surface of the labrum, known as epipharynx, appears smooth and possesses fewer sensilla compared to its outer surface, including Sco1, Sco2, and Sca (Figure 3E,F). Notably, these sensilla are surrounded by diverse types of cuticle protrusions (e.g., spiny processes, palmate processes, and scaly processes) (Figure 3G,H).

### 3.5. Mandible

**Figure 4:**
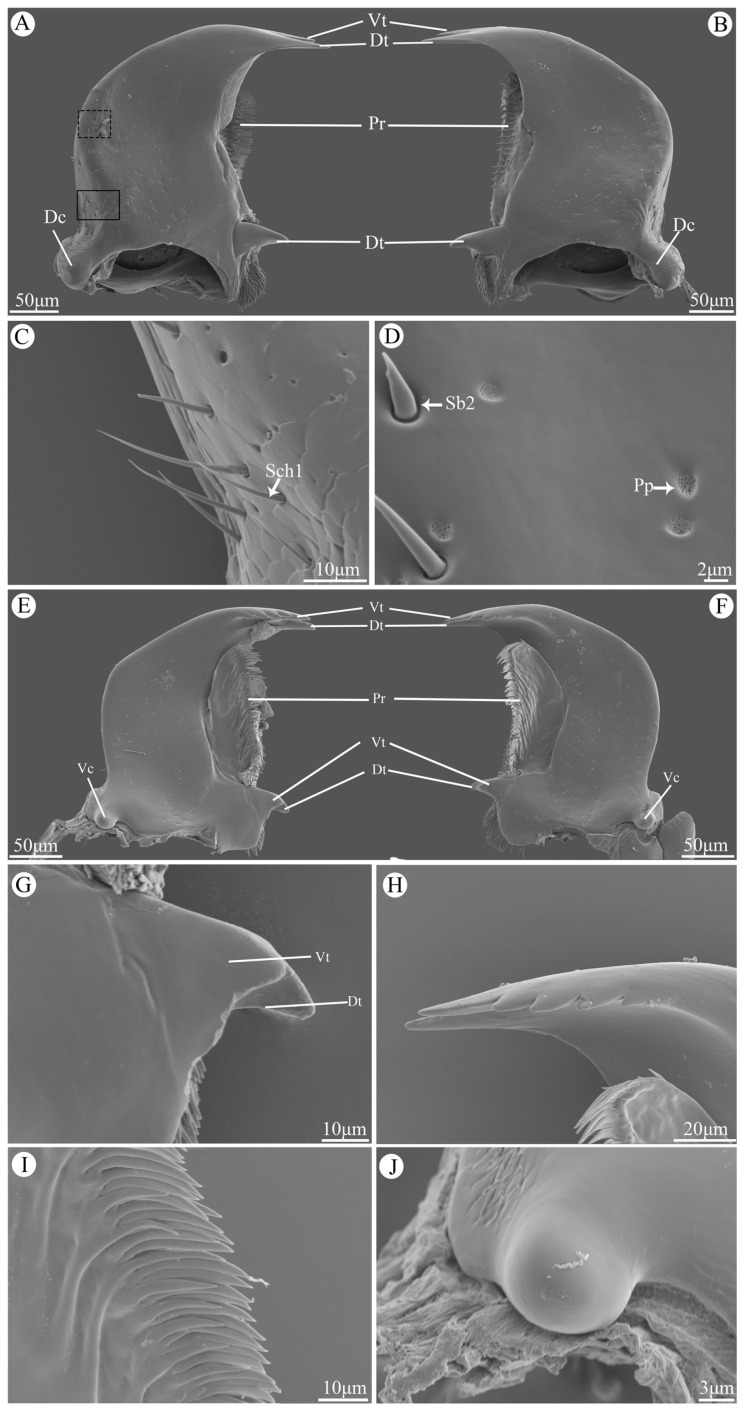
Scanning electron micrographs of the mandible of *Vibidia duodecimguttata*. (**A**) Dorsal view of the left mandible; (**B**) Dorsal view of the right mandible; (**C**) Enlarged view of black solid box in (**A**); (**D**) Enlarged view of black dashed box in (**A**); (**E**) Ventral view of right mandible; (**F**) Ventral view of left mandible; (**G**) Enlarged view of dorsal teeth (Dt) and ventral teeth (Vt); (**H**) Enlarged view of ventral of incisor; (**I**) Enlarged view of ventral view of prostheca; (**J**) Enlarged view of ventral condyles (Vt). Dt: dorsal teeth; Vt: ventral teeth; Pr: prostheca; Dc: dorsal condyles; Vc: ventral condyles; Sch1: sensilla chaetica I; Pp: perforated plates; Sb2: sensilla basiconica II.

The mandibles are highly sclerotized and symmetrically distributed beneath the labrum. Their surfaces are relatively smooth with some Cp on the central part and Sch1, Sb2, and Pp in the marginal region (Figure 4C,D). The apical incisor region consists of two teeth, namely dorsal teeth and ventral teeth. The ventral teeth are smooth on the dorsal side but serrated on the ventral side with four little accessory teeth (Figure 4H). The prostheca is located centrally on the ventral side of the mandibles, and it possesses numerous multi-layered slender bristles along its margin (Figure 4I). The molar region consists of two teeth, but their characteristics differ between left and right mandible. On the right mandible, the ventral tooth is slightly smaller and blunter compared to its dorsal counterpart (Figure 4G), whereas both teeth appear similar in size and shape on the left mandible (Figure 4F). The dorsal condyle exhibits an ellipsoidal shape covered with scaly processes (Figure 4A,B), while a globular structure characterizes the smooth surface of the ventral condyle (Figure 4J).

### 3.6. Maxillae

**Figure 5:**
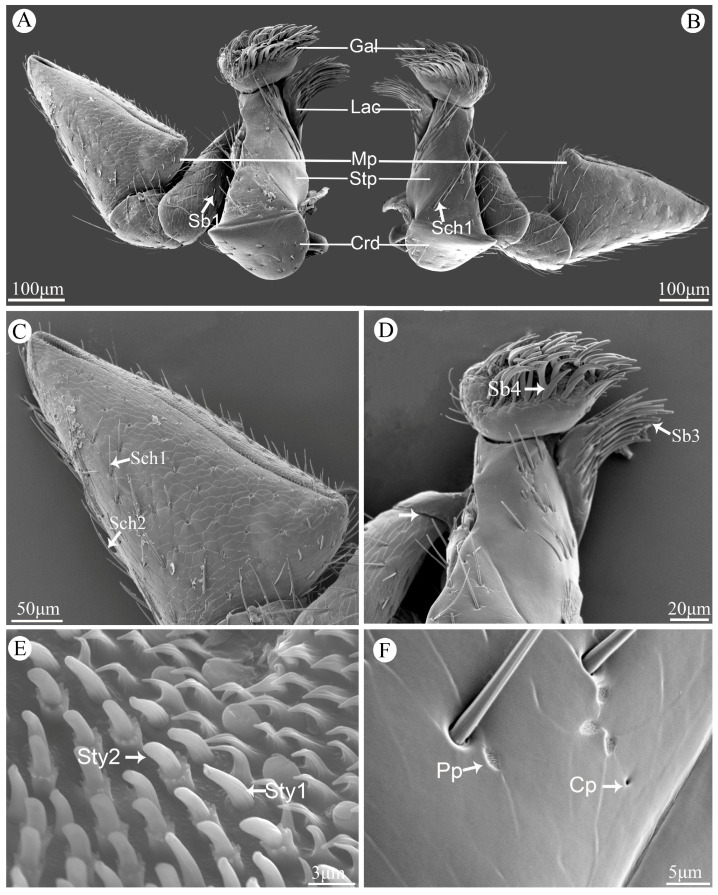
Scanning electron micrographs of the maxillae of *Vibidia duodecimguttata*. (**A**) Ventral view of right maxillae; (**B**) Ventral view of left maxillae; (**C**) Enlarged view of maxillary palp; (**D**) Enlarged view of galea and lacinia; (**E**) Sensilla styloconica I (Sty1) and sensilla styloconica II (Sty2); (**F**) Perforated plates (Pp) and culticular pores (Cp). Crd: cardo; Stp: Stipes; Gal: galea; Lac: lacinia; Mp: maxillary palps; Sch1: sensilla chaetica I; Sch2: sensilla chaetica II; Sb1: sensilla basiconica I; Sb3: sensilla basiconica III; Sb4: sensilla basiconica IV.

The maxillae are paired and symmetrical structures, and each composed of the cardo, stipe, lacinia, galea, and maxillary palp (Figure 5A,B). The cardo is hemispherical and articulates with the head. The trapezoidal stipe bears Sch1 and Cp. Situated distally on the maxillae is a spoon-like structure called the galea, which possesses abundant Sch1 and Sb4 on its upper surface. The lacinia resembles a brush-like structure with numerous Sb3 along its margin (Figure 5D).

Comprising four segments, the maxillary palp exhibits distinct characteristics in each segment. The first segment is relatively small and attaches to the stipe. Covered by a small amount of Sb1, the second segment assumes a rectangular shape. Fan-shaped in appearance, the third segment precedes an enlarged final segment that takes on an isosceles triangle form (Figure 5C). All segments of the maxillary palpi possess scaly surfaces covered by Sch1, Sch2, Sb1, Pp, and Cp (Figure 5C,F), while densely distributed sensilla and cuticular processes dominate in sensory regions at the apex of this last segment of maxillary palp; notable types include Sty1 and Sty2 (Figure 5E).

### 3.7. Labium

**Figure 6:**
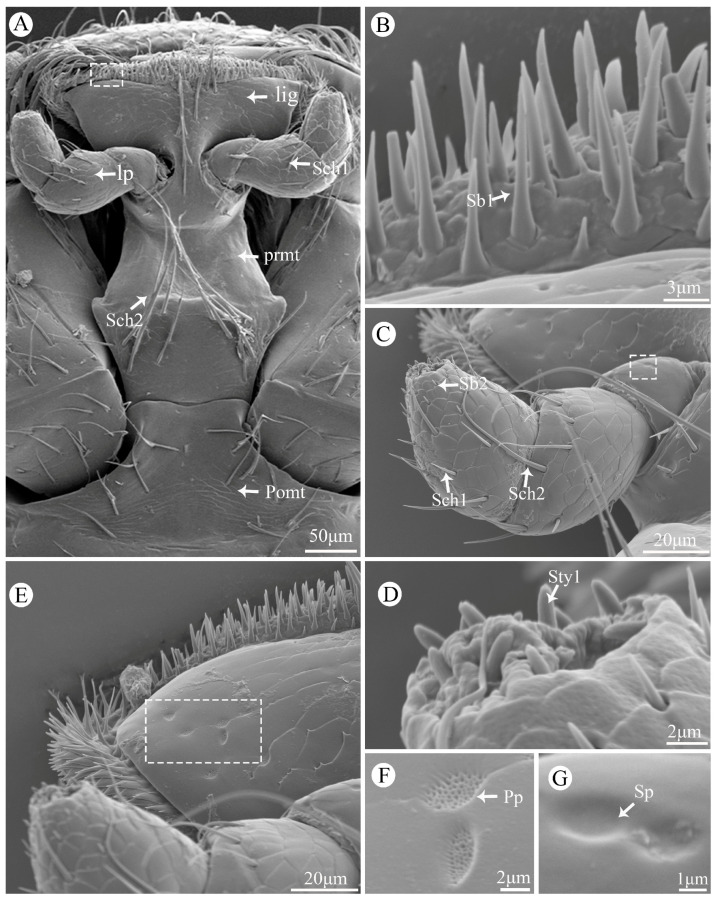
Scanning electron micrographs of the labium of *Vibidia duodecimguttata*. (**A**) Ventral view of labium; (**B**) Enlarged view of white dashed box in (**A**); (**C**) Enlarged view of labial palp; (**D**) Enlarged view of labial palp tip; (**E**) Enlarged view of ligula; (**F**) Enlarged view of white dashed box in (**E**); (**G**) Enlarged view of white dashed box in (**C**). Lig: ligula; Lp: labial palp; Prmt: prementum; Pomt: postmentum; Sch1: sensilla chaetica I; Sch2: sensilla chaetica II; Sb1: sensilla basiconica I; Sb2: sensilla basiconica II; Pp: perforated plates; Cp: culticular pores; Sty1: sensilla styloconica I; Sp: sensilla placodea.

The labium consists of a postmentum, prementum, ligula, and a pair of labial palpi (Figure 6A). The postmentum is hatchet-shaped with a smooth surface and covered with several Sch1 on the marginal region (Figure 6A). The prementum is wide in the middle and narrow at both ends, with its surface covered by Sch1, Sch2, and Cp (Figure 6A). The labial palp comprises three segments. The first segment has a smooth surface with only Sp present (Figure 6C,G); the second segment is conical with scaly processes on its surface and covered by Sch2 (Figure 6C). The last segment is bullet-shaped, covered by Sch1 on its surface while Sb2 covers the extremity around part (Figure 6C), and numerous Sty1 are distributed on top (Figure 6D). The ligula is trapezoidal, and its top region is covered by Sb1 (Figure 6B), Sch1, Pp (Figure 6E,F), and Cp.

## 4. Discussion

### 4.1. The Morphology of Mouthparts

In this study, the mouthparts of Vibidia duodecimguttata are typical chewing mouthparts like other ladybeetles. However, the specific structures of the mouthparts have exhibited different morphology to adapt to different diets [8,10,29,30]. However, in the same diet, their food resources maybe different. For example, predatory ladybeetles may feed on aphids, scale insects, whiteflies, and mites. Therefore, their mouthpart structures will evolve into different forms based on different diets and food resources [31]. Moreover, the morphology of the mouthparts structures in the same subfamily or genus may also be different [32]. For example, a detailed comparative analysis between the mouthparts of *Hippodamia variegata* and *Coccinella transversoguttata*, which belong to the same subfamily, revealed numerous differences in the type of sensilla and the shape of a specific part of the mouthparts [20]. Although they are in the same genus and have similar food resource, Coccinella transversalis possesses a wider labrum and more types of sensilla than Coccinella septempunctata [9].

The morphology of the mandible was reported to be determined by the feeding method [29], and as a consequence, the mandible is the key to distinguishing between different feeding habits of ladybeetles [31]. Predatory ladybeetles have either bicuspids or unidentate apex of mandibles used for piercing the prey and sucking out the juice [31]. For phytophagous species, their mandibles have multiple denticles that are used to scrape the surface of the leaves and ingest plant sap. For mycetophagous species, however, mandibles have several accessory teeth on the ventral teeth that are quite helpful in the process of collecting fungal spores. The mandibles of V. duodecimguttata in this study are similar to the other mycetophagous ladybeetles. The incisors of the mandible bear two teeth, the dorsal one is much bigger, while the ventral tooth bears a row of accessory teeth. As reported in Illeis chinensis, there is only one accessory tooth on the dorsal tooth of the incisor, while 12–16 accessory teeth can be found on the ventral tooth [21]. *Psyllobora vigintiduopunctata* have only three accessory teeth [31], whereas *V. duodecimguttata* in this study have four accessory teeth. This difference in the number of accessory teeth may be related to their feeding habitat or the type of spores.

In phytophagous and predatory species, the comb-like prostheca are used to transport plant sap and empty ‘skin’ [33], while in mycetophagous species, the shorter but denser comb-like prostheca are used to collect spores from the incisor region to the molar region [31,33,34].

In addition to the mandibles, the morphology of the other parts of the mouthparts is also related to feeding habits. Compared to ladybeetles with other diets, the labrum, maxillae, and labium of the mycetophagous ladybeetles V. duodecimguttata are in agreement with the morphology of the mouthpart of I. chinensis. Their labrum are enlarged on two sides of the middle part, which may better protect the other structures of the mouthparts. The ligula of the labium is broader, which may better enclose the mandibles, allowing more fungal spores to be taken in, and preventing them from flowing out better [21]. Secondly, in comparison to phytophagous and predatory species, mycetophagous ladybeetles demonstrate a distinct expansion of the sensory field on the terminal segment of their maxillary palpi, potentially augmenting their capacity to efficiently locate and access food sources with greater precision. Additionally, the curved setae of the maxillary galea and lacinia exhibit exceptional suitability for efficient spore and conidia release as well as collection [35].

### 4.2. The Function of Sensilla

The numerous sensilla distributed on the mouthparts serve various functions in feeding, host, and mate detection, etc. [36]. During feeding, maxillary palpi and labial palpi play a crucial role in gustatory and olfactory perception [37], with a significant number of sensilla located in the sensory field on the distal region of these organs [20]. The predominant types of sensilla found on both maxillary palpi and labial palpi of *V. duodecimguttata* are consistent with those observed in other ladybeetles: Sty1 and Sty2 at the tip of the maxillary palpi, as well as Sty1 at the end of labial palpi. Sty2 is characterized by substantial apical pores as reported in other ladybeetles [13,21], and in conjunction with the foraging and feeding behavior of the maxillary palpi [38], Sty2 has been recognized as an olfactory sensillum [9,13,20,39], whereas Sty1 has been associated with gustatory and mechanical functions [37]. Several potential roles for olfaction can be hypothesized including habitat selection, spawning site localization, prey detection, or intersexual communication. Olfactory sensilla present on mouthparts primarily contribute to prey search activities [40]. In addition, similar to *I. chinensis*, *V. duodecimguttata* has only one Sp on labial palp, and this type of sensilla has been reported to detect the cuticular stress exerted on palp in the process of prey capture and feeding [41].

Two types of glandular structures were identified on the mouthparts of *V. duodecimguttata*, namely Cp and Pp. Cp represents a common type of stomata in ladybeetles, widely distributed on the surface of the mouthparts, serving as terminal apparatus for secretory cells [42]. On the other hand, Pp is also extensively distributed across all surfaces of the mouthparts, characterized by numerous perforated plates on the dorsal side of the maxillae that facilitate drainage of glandular cells onto the cuticular surface [43]. Despite their resemblance to sensory organs [30], our study observed abundant secretions emanating from these pores, confirming their glandular nature rather than being sensory organs.

The other sensilla identified in this study exhibit functional similarities to those commonly observed in other insects, serving as mechano-, temperature- or humidity-reception [44]. Sensilla chaetica, as a prominent mechanoreceptor on the mouthparts, detects external stimuli [38,45]. Sensilla basiconica is the second most abundant sensilla and is usually involved in taste perception and food detection [38,46,47], However, sensilla coeloconic and sensilla campaniformia are exclusively located on the surface of epipharynx in *V. duodecimguttata*. Sensilla coeloconica play a role in chemoreception while also sensing changes in temperature and humidity [15,38,43], whereas sensilla campaniformia function as cuticular strain detectors that respond to tension and strain within the cuticle when it comes into contact with food [48,49,50,51].

### 4.3. Difference Between Mycetophagous Ladybeetle V. duodecimguttata and I. chinensis

As the sole reported mycetophagous ladybeetles species studied on mouthpart morphology, *I. chinensis* mainly feeds on hyphae and spores of powdery mildew affecting crops and fruit trees. By contrast, *V. duodecimguttata* in this study primarily consumes fungal spores found on broad-leaved trees and shrubs [22]. The fine morphology of the mouthparts of these two species may be different on account of different feeding environments.

By comparing with *I. chinensis*, the first and most significant difference lies in the type of sensilla: both sensilla digitiformia and böhm bristles are notably absent in *V. duodecimguttata*. Sensilla digitiformia serve as receptors for heat, water, or carbon dioxide stimuli and also have been shown to play a role as tactile mechanoreceptors capable of detecting contact and vibration stimuli [52]. On the other hand, böhm bristles respond to external stimuli associated with gravity perception. Schneider’s study on insect antennae revealed that böhm bristles on the antennae responded to gravity stimulation when all other body joints were mechanically fixed [53]. Therefore, we hypothesize that *V. duodecimguttata* may rely more heavily on antennal sensilla for heat, water, or carbon dioxide reception and gravity stimulation. Further research and discussion regarding the antennal sensilla of *V. duodecimguttata* are necessary to substantiate these claims.

The other significant difference between these two ladybeetles was found on the maxillary palpi, especially the terminal region. In *I. chinensis*, the terminal of the maxillary palpi expanded in a fan-shaped manner, whereas in *V. duodecimguttata* it assumed a triangular shape, from which we speculated that the fan shape may allow for greater range of motion to allow for better search and perception.

Furthermore, the sensilla on the mouthpart of *I. chinensis* is more abundant than that of *V. duodecimguttata*, especially on the maxillary palpi and labial surface. The surface of the maxillary palpi of *I. chinensis* was found to be covered with an increased number of Sch1, and a few Sch3 were present on the prementum of their labium; additionally, there were 28 Sty1 at the end of the labial palpi in *I. chinensis* as opposed to only about 14 Sty1 in *V. duodecimguttata*.

The dorsal and ventral teeth of the mandible in *I. chinensis* are much sharper [21]. Additionally, the ligula in *I. chinensis* is broader, and the labial palpi are longer compared to other species [21]. These morphological variations may be attributed to specific ecological adaptions and evolutionary processes.

## 5. Conclusions

This study provides a detailed examination of the fine morphology of each component of the mouthpart of *V. duodecimguttata*, while also investigating the type, distribution, and morphological character of the sensilla on these mouthparts. As one of the few reports on mouthpart morphology in mycetophagous ladybeetle species, we summarize both similarities and differences observed in comparison to published species, thereby establishing a solid foundation for further research into inter-diet comparisons. Future studies should focus on exploring sensory functions to elucidate variations in sensilla among different species and diets, ultimately shedding light on the feeding mechanisms employed by mycetophagous ladybeetles.

## Figures and Tables

**Table 1:** Morphological characters of sensilla and glandular structures on the mouthparts of *Vibidia duodecimguttata*.

Type		Shape	Socket	Surface	Pore	Length (μm)	Diameter (μm)	Distribution	Fixed Position
Sensilla	Sch1	Peg	Concave	Grooved	No	50.3–59.6	2.2–3.2	Lm, Md, Mx, Lb	No
	Sch2	Hair, Peg	Concave	Grooved	No	93.2–115.3	2.1–3.9	Lm, Mx, Lb	No
	Sb1	Coniform	Concave	Smooth	No	8.1–13.0	1.7–2.0	Lm, Mx, Lb	No
	Sb2	Coniform	Concave	Smooth	No	2.4–4.2	0.7–1.7	Md, Lb	No
	Sb3	Hair, Cylindrical	Concave	Smooth	No	39.7–50.8	1.9–4.2	Lac	Yes
	Sb4	Falcate	Convex	Smooth	No	32.4–40.3	3.1–5.4	Gal	Yes
	Sty1	Conical	Convex	Grooved	Apical pore	2.8–3.7	0.6–1.3	Mp, Lp	Yes
	Sty2	Cylindrical	Convex	Grooved	Apical pore	1.7–2.9	0.6–1.1	Mp	Yes
	Sca	Round	Convex	Papilliform	Multiporous	-	3.4–5.1	Epi	Yes
	Sco1	Coniform	Convex	Smooth	No	-	1.4–1.8	Epi	Yes
	Sco2	Coniform	Convex	Rugose	No	3.2–5.0	3.2–4.7	Epi	Yes
	Sp	Round	Concave	Smooth	No	–	1.6–2.8	Lp	Yes
Glandular structures	Pp	Irregular	Concave	No	Multiporous	-	0.4–0.8	Lm, Md, Mx, Lb	No
	Cp	Hole	Concave	-	Uniporous	-	1.7–4.6	Lm, Md, Mx, Lb	No

Sch: sensilla chaetica; Sb: sensilla basiconica; Sty: sensilla styloconica; Sco: sensilla coeloconica; Sca: sensilla campaniformia; Sp: sensilla placodea; Pp: perforated plates; Cp: cuticular pores; Lm: labrum; Md: mandible; Mx: maxillae; Lb: labium; Mp: maxillary palp; Lp: labial palp; Epi: epipharynx; Gal: galea; Lac: lacinia.

## Data Availability

The data presented in this study are available in article.
